# *In vitro* activity of ozenoxacin against *Staphylococcus aureus* and *Streptococcus pyogenes* clinical isolates recovered in a worldwide multicentre study (2020–2022)

**DOI:** 10.1093/jacamr/dlae088

**Published:** 2024-06-13

**Authors:** María García-Castillo, Marta Hernández-García, Adriana Correa, Marco Coppi, Thomas Griener, Thomas Fritsche, Cristina Pitart, Jorge Sampaio, Harald Seifert, Karen Wake, Mandy Wootton, Jordi Vila, Rafael Cantón

**Affiliations:** Servicio de Microbiología, Hospital Universitario Ramón y Cajal, Madrid, Spain; CIBER de Enfermedades Infecciosas (CIBERINFEC), Instituto Salud Carlos III, Madrid, Spain; Servicio de Microbiología, Hospital Universitario Ramón y Cajal, Madrid, Spain; CIBER de Enfermedades Infecciosas (CIBERINFEC), Instituto Salud Carlos III, Madrid, Spain; Clínica Imbanaco Grupo Quirón Salud, Cali, Valle del Cauca, Colombia; Universidad Santiago de Cali, Cali, Valle del Cauca, Colombia; Department of Experimental and Clinical Medicine, University of Florence, Florence, Italy; Microbiology and Virology Unit, Florence Careggi University Hospital, Florence, Italy; Clinical Section of Microbiology, Alberta Precision Laboratories, Calgary, Alberta, Canada; Department of Pathology & Laboratory Medicine, Cumming School of Medicine, University of Calgary, Calgary, Alberta, Canada; Division of Laboratory Medicine, Marshfield Clinic Research Institute, Marshfield, WI, USA; CIBER de Enfermedades Infecciosas (CIBERINFEC), Instituto Salud Carlos III, Madrid, Spain; Servicio de Microbiología, Hospital Clinic, Barcelona, Spain; Department of Clinical and Toxicological Analyses, School of Pharmacy, University of São Paulo, São Paulo, Brazil; German Center for Infection Research (DZIF), Partner Site Bonn-Cologne, Cologne, Germany; Institute of Translational Research, CECAD Cluster of Excellence, University of Cologne, Cologne, Germany; Canadian Antimicrobial Resistance Alliance, Health Sciences Centre University of Manitoba, Manitoba, Canada; Specialist Antimicrobial Chemotherapy Unit, Public Health Wales, University Hospital of Wales, Cardiff, UK; CIBER de Enfermedades Infecciosas (CIBERINFEC), Instituto Salud Carlos III, Madrid, Spain; Servicio de Microbiología, Hospital Clinic, Barcelona, Spain; Servicio de Microbiología, Hospital Universitario Ramón y Cajal, Madrid, Spain; CIBER de Enfermedades Infecciosas (CIBERINFEC), Instituto Salud Carlos III, Madrid, Spain

## Abstract

**Objectives:**

We performed a multicentre study (2020–2022) to compare the *in vitro* activity of ozenoxacin and comparator agents against *Staphylococcus aureus* and *Streptococcus pyogenes* clinical isolates from skin and soft-tissue infections (SSTI).

**Methods:**

A total of 1725 isolates (1454 *S. aureus* and 271 *S. pyogenes*) were collected in 10 centres from eight countries between January 2020 and December 2022. Antimicrobial susceptibility testing was determined (microdilution-SENSITITRE). Results were interpreted following European Committee on Antimicrobial Susceptibility Testing (EUCAST) 2023 (clinical breakpoints, ECOFF) and CLSI criteria.

**Results:**

Ozenoxacin exhibited high *in vitro* activity against *S. aureus* (MIC_50/90_ = 0.002/0.12 mg/L) and *S. pyogenes* (MIC_50/90_ = 0.015/0.03 mg/L), inhibiting 99% of the isolates at MIC ≤ 0.5 mg/L and at MIC ≤ 0.06, respectively. The most active comparators against *S. aureus* were retapamulin (MIC_90_ = 0.12 mg/L), fusidic acid (MIC_90_ = 0.25 mg/L) and mupirocin (MIC_90_ = 0.5 mg/L); and against *S. pyogenes* were retapamulin (MIC_90_ = 0.03 mg/L), clindamycin (MIC_90_ = 0.12 mg/L) and mupirocin (MIC_90_ = 0.25 mg/L). Ciprofloxacin and methicillin resistant rates for *S. aureus* were 31.3% (455/1454) and 41% (598/1454), respectively. Additionally, 62% (373/598) of the MRSA were also ciprofloxacin non-susceptible, whereas only 10% (23/271) of the MSSA were ciprofloxacin resistant. Ozenoxacin was more active against ciprofloxacin-susceptible *S. aureus* than against ciprofloxacin-resistant isolates, and showed a slightly higher MIC in MRSA isolates than in MSSA. However, ozenoxacin activity was comparable in both ciprofloxacin-resistant MSSA and MRSA subsets. On the other hand, ozenoxacin had similar activity in ciprofloxacin-susceptible and resistant *S. pyogenes* isolates.

**Conclusions:**

Ozenoxacin is a potent antimicrobial agent of topic use against Gram-positive bacteria causing SSTI, including MRSA isolates non-susceptible to ciprofloxacin.

## Introduction

*Staphylococcus aureus* and *Streptococcus pyogenes* are major causative bacteria responsible for skin and soft-tissue infections (SSTIs) such as impetigo.^1,2^ Clinical guidelines recommend the use of topical antibacterial agents for localized impetigo, reserving oral or even intravenous antibiotics for those cases involving extensive lesions resistant to topical therapy, systemic infections and the management of outbreaks.^3^ Mupirocin, fusidic acid and more recently retapamulin, are the topical antimicrobials more frequently used to treat impetigo in clinical practice.^4–6^ However, antimicrobial resistance to these topical compounds has been reported, possibly due to the increasing incidence of community-acquired MRSA infections, posing a further limitation to the overall efficacy of available antibiotics.^7–11^

Ozenoxacin is a novel non-fluorinated quinolone antibacterial that was approved in 2017 in the EU, Canada and the USA for the topical treatment of impetigo in patients ≥2 months of age.^12–14^ Ozenoxacin has showed excellent *in vitro* activity against Gram-positive bacteria causing SSTIs, including *S. aureus* and *S. pyogenes* clinical isolates.^12,15–17^ Previous studies demonstrated higher efficacy of ozenoxacin compared to other topical antibacterial agents, especially in cases involving quinolones-resistant MRSA isolates.^16–18^

Surveillance studies of antimicrobial resistance are necessary for monitoring changes in resistance rates, detecting the emergence and spread of new resistances, assessing the scale of the resistance problem at a local, national or international level, and providing data for selection of empirical therapy. The purpose of this study was to evaluate the *in vitro* activity of ozenoxacin and comparator agents against the most prevalent bacterial pathogens that concern SSTI (*S. aureus* and *S. pyogenes*) prospectively collected in eight countries from patients with impetigo or community-acquired skin infections between 2020 and 2022.

## Methods

### Study design and bacterial isolates

A multicentre study was designed to assess the *in vitro* activity of ozenoxacin and comparator agents against Gram-positive clinical isolates prospectively recovered in 10 centres from eight countries [Spain (*n* = 2), Canada (*n* = 2), Brazil (*n* = 1), Colombia (*n* = 1), Germany (*n* = 1), Italy (*n* = 1), UK (*n* = 1), USA (*n* = 1)] between January 2020 and December 2022. A total of 1725 isolates [*S. aureus* (*n* = 1454) and *S. pyogenes* (*n* = 271)] were recovered from paediatric and adult patients with uncomplicated (uSSTIs) and/or complicated SSTI (cSSTIs), including impetigo. Centres were asked to collect up to 50 *S. aureus* (25 MSSA and 25 MRSA) and 20 *S. pyogenes*. Overall, 650 isolates were collected from eight centres in 2020, 573 isolates from nine centres in 2021 and 502 isolates from seven centres in 2022. Only one isolate per patient was included. All isolates were sent to the coordinator laboratory (Hospital Universitario Ramón y Cajal, Madrid, Spain) for further microbiological studies. Distribution of isolates by microorganism and year of collection is summarized in Table S1 (available as Supplementary data at *JAC-AMR* Online). Species identification was carried out at each participant hospital and confirmed at the coordinating laboratory using MALDI-TOF mass spectrometry (Bruker-Daltonics, Bremen, Germany). The ethics committee of the coordinating centre approved the study (ref. 336-19).

### Antimicrobial susceptibility testing

Susceptibility of ozenoxacin and comparators was determined by broth microdilution using Trek (Thermo Fisher Scientific, East Grinstead, West Sussex, UK) 96-well panels. MIC_50_ and MIC_90_ values were also calculated for each antimicrobial agent. The tested antimicrobial concentration ranges were as follows: penicillin, 0.03–0.5 mg/L; ozenoxacin, 0.001–4 mg/L; ciprofloxacin, 0.06–16 mg/L; levofloxacin; 0.06–16 mg/L; mupirocin, 0.06–256 mg/L; fusidic acid, 0.03–16 mg/L; vancomycin, 0.25–2 mg/L; erythromycin, 0.06–16 mg/L; clindamycin, 0.015–16 mg/L and retapamulin, 0.015–2 mg/L. In addition, cefoxitin as marker for *mecA*-mediated methicillin resistance (in a single concentration, 4 mg/L) was included in the panel following the recommendations of the CLSI. *S. aureus* ATCC 29213 and ATCC 43300 were used for quality control.^19^ The results were interpreted in accordance with the European Committee on Antimicrobial Susceptibility Testing (EUCAST) (EUCAST-2023, https://www.eucast.org/clinical_breakpoints) and CLSI (CLSI-2020, https://clsi.org/standards/products/microbiology/documents/m100/) guidelines.^19,20^ No breakpoints are yet defined by the CLSI or EUCAST for ozenoxacin. For retapamulin, EUCAST epidemiological cut-off (ECOFF) values were used.

## Results

### Bacterial isolates

A total of 1454 *S. aureus* (84.3%) and 271 *S. pyogenes* (15.7%) isolates were recovered from 209 paediatric (12.1% ≤ 18 years, range: 5 days–18 years) and 1481 adult (85.9% > 18 years, range: 18–101 years) patients. Demographic information was not available for 35 patients. All patients had community-acquired skin infections [*S. aureus* (*n* = 1072, 62.1%) and *S. pyogenes* (*n* = 208, 12.1%)], including impetigo [*S. aureus* (*n* = 19, 1.1%) and *S. pyogenes* (*n* = 5, 0.3%)] or other skin infections [*S. aureus* (*n* = 363, 21.0%) and *S. pyogenes* (*n* = 58, 3.4%)]. Isolates were recovered from wounds (*n* = 708, 41.0%), abscesses (*n* = 324, 18.8%), skin (*n* = 353, 20.5%), soft tissues (*n* = 205, 11.9%) and not specified skin and soft-tissue samples (*n* = 135, 7.8%). Distribution of species by type of infection and sample source is summarized in Table S2.

Of the 1454 *S. aureus* isolates included in the study, 598 (41.1%) were MRSA and 856 (58.9%) were MSSA. According to the EUCAST criteria, more than a half (62.4%, 373/598) of the MRSA were also ciprofloxacin non-susceptible, whereas only 9.6% (82/856) of the MSSA were resistant to ciprofloxacin. Among the *S. pyogenes* isolates, 8.5% (23/271) were resistant to ciprofloxacin.

### *Ozenoxacin* in vitro *activity in* S. aureus

**Figure 1.**
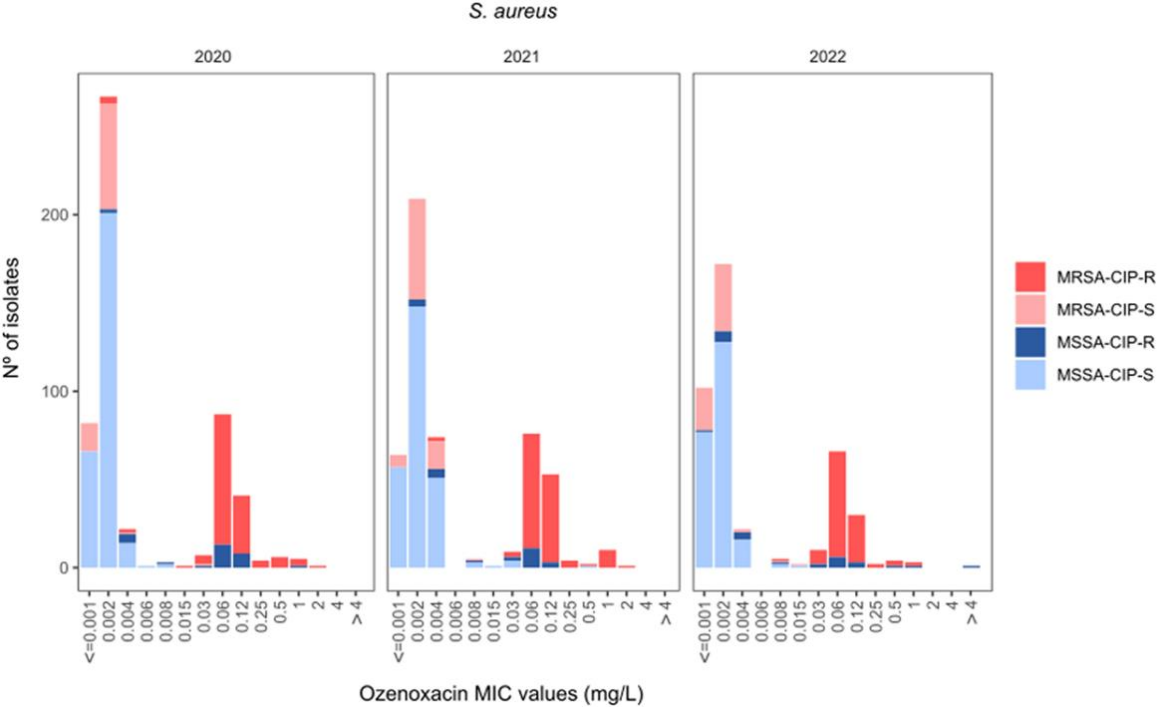
Distribution per year of the *S. aureus* isolates (2020, *n* = 527; 2021, *n* = 508; 2022, *n* = 419) by the ozenoxacin MIC value and the ciprofloxacin and methicillin susceptibility. MRSA, methicillin-resistant *S. aureus*; MSSA, methicillin-susceptible *S. aureus*. Ciprofloxacin MIC values were interpreted as susceptible (*S* ≤ 0.001 mg/L; *I* = 0.001–1 mg/L) and resistant (*R* > 1 mg/L) (EUCAST-2023).

Ozenoxacin demonstrated excellent overall activity (MIC_50/90_ = 0.002/0.12 mg/L, range ≤0.001–>4 mg/L) against all 1454 *S. aureus* isolates, inhibiting 98.6% at a MIC of ≤0.5 mg/L. The susceptibility of comparator agents according to EUCAST was as follows: vancomycin, 99.7%; retapamulin, 99.2%; mupirocin, 94.6%; fusidic acid, 93.3%; clindamycin, 89.2% and erythromycin, 53.8% (Table 1). Overall, changes were not observed in the ozenoxacin MIC values over time: MIC_50/90_ = 0.002/0.12 mg/L, range ≤0.001–2 mg/L, during 2020; MIC_50/90_ = 0.002/0.12 mg/L, range ≤0.001–2 mg/L, during 2021 and MIC_50/90_ = 0.002/0.06 mg/L, range ≤0.001–>4 mg/L, during 2022 (Table 2, Figure 1). Summary MIC data and interpretative susceptibility results for ozenoxacin against all *S. aureus* by methicillin and ciprofloxacin susceptibilities during the 3 years of study are shown in Table 2.

**Table 1.** Antimicrobial activity of ozenoxacin and comparators tested against *S. aureus* by methicillin and ciprofloxacin susceptibility

	MIC (mg/mL)			EUCAST						CLSI					
				S		I		R		S		I		R	
	MIC_50_	MIC_90_	Range	*n*	%	*n*	%	*n*	%	*n*	%	*n*	%	*n*	%
All *S. aureus* (*n* = 1454)															
Penicillin	>0.5	>0.5	≤0.03–>0.5	219	15.1	—	—	1235	84.9	219	15.1	—	—	1235	84.9
Cefoxitin^a^	≤4	>4	—	856	58.9	—	—	598	41.1	—	—	—	—	—	—
Ozenoxacin	0.002	0.12	≤0.001–>4	—	—	—	—	—	—	—	—	—	—	—	—
Vancomycin	1	1	≤0.25–>2	1450	99.7	—	—	4	0.3	1450	99.7	4	0.3	—	—
Ciprofloxacin	0.5	>16	≤0.06–>16	—	—	999	68.7	455	31.3	999	68.7	33	2.3	422	29.0
Levofloxacin	0.25	>16	≤0.06–>16	—	—	1034	71.1	420	28.9	1034	71.1	4	0.3	416	28.6
Mupirocin^a^	0.25	0.5	≤0.06–>256	1376	94.6	—	—	78	5.4	—	—	—	—	—	—
Fusidic acid	0.12	0.25	≤0.03–>16	1356	93.3	—	—	98	6.7	—	—	—	—	—	—
Erythromycin	0.25	>16	≤0.06–>16	782	53.8	-	−	672	46.2	775	53.3	34	2.3	645	44.4
Clindamycin	0.12	4	≤0.015–>16	1297	89.2	-	-	157	10.8	1301	89.5	5	0.3	148	10.2
Erythromycin/clidamycin	≤1/0.5	>8/1.5	≤1/0.5–>8/1.5	—	—	—	—	—	—	—	—	—	—	—	—
Retapamulin^a^	0.06	0.12	≤0.015–>2	1442	99.2	-	-	12	0.8	—	—	—	—	—	—
All *S. aureus* ciprofloxacin resistant (*n* = 455)															
Penicillin	>0.5	>0.5	≤0.03–>0.5	19	4.2	—	—	436	95.8	19	4.2	—	—	436	95.8
Cefoxitin^a^	>4	>4	>4	82	18.0	—	—	373	82.0	—	—	—	—	—	—
Ozenoxacin	0.06	0.12	≤0.001–>4	—	—	—	—	—	—	—	—	—	—	—	—
Vancomycin	1	1	≤0.25–>2	452	99.3	—	—	3	0.7	452	99.3	3	0.7	—	—
Ciprofloxacin	8	>16	2–>16	—	—	0	0.0	455	100.0	0	0	33	7.3	422	92.7
Levofloxacin	8	>16	≤0.06–>16	—	—	36	7.9	419	92.1	36	7.9	4	0.9	415	91.2
Mupirocin^a^	0.25	16	≤0.06–>256	396	87.0	—	—	59	13.0	—	—	—	—	—	—
Fusidic acid	0.12	0.25	≤0.03–>16	425	93.4	—	—	30	6.6	—	—	—	—	—	—
Erythromycin	>16	>16	≤0.06–>16	130	28.6	—	—	325	71.4	127	27.9	11	2.4	317	69.7
Clindamycin	0.12	>16	≤0.015–>16	343	75.4	—	—	112	24.6	344	75.6	2	0.4	109	24.0
Erythromycin/clidamycin	≤1/0.5	>8/1.5	≤1/0.5–>8/1.5	—	—	—	—	—	—	—	—	—	—	—	—
Retapamulin^a^	0.06	0.12	≤0.015–>2	448	98.5	—	—	7	1.5	—	—	—	—	—	—
All *S. aureus* ciprofloxacin susceptible (*n* = 999)															
Penicillin	>0.5	>0.5	≤0.03–>0.5	200	20.0	—	—	799	80.0	200	20.0	—	—	799	80.0
Cefoxitin^a^	≤4	>4	≤4–>4	774	77.5	—	—	225	22.5	—	—	—	—	—	—
Ozenoxacin	0.002	0.004	≤0.001–0.5	—	—	—	—	—	—	—	—	—	—	—	—
Vancomycin	1	1	≤0.25–>2	998	99.9	—	—	1	0.1	998	99.9	1	0.1	—	—
Ciprofloxacin	0.25	0.5	≤0.06–1	—	—	999	100.0	0	0	999	100.0	0	0	0	0
Levofloxacin	0.25	0.25	≤0.06–8	—	—	998	99.9	1	0.1	998	99.9	0	0	1	0.1
Mupirocin^a^	0.25	0.25	≤0.06–>256	980	98.1	—	—	19	1.9	—	—	—	—	—	—
Fusidic acid	0.12	0.25	≤0.03–>16	931	93.2	—	—	68	6.8	—	—	—	—	—	—
Erythromycin	0.25	>16	≤0.06–>16	652	65.3	—	—	347	34.7	648	64.9	23	2.3	328	32.8
Clindamycin	0.12	0.12	≤0.015–>16	954	95.5	—	—	45	4.5	957	95.8	3	0.3	39	3.9
Erythromycin/clidamycin	≤1/0.5	>8/1.5	≤1/0.5–>8/1.5	—	—	—	—	—	—	—	—	—	—	—	—
Retapamulin^a^	0.06	0.12	≤0.015–>2	994	99.5	—	—	5	0.5	—	—	—	—	—	—
All MRSA (*n* = 598)															
Penicillin	>0.5	>0.5	≤0.03–>0.5	4	0.7	—	—	594	99.3	4	0.7	—	—	594	99.3
Cefoxitin^a^	>4	>4	>4	0	0	—	—	598	100	—	—	—	—	—	—
Ozenoxacin	0.06	0.12	≤0.001–2	—	—	—	—	—	—	—	—	—	—	—	—
Ciprofloxacin	8	>16	0.12–>16	—	—	225	37.6	373	62.4	225	37.6	8	1.3	365	61.0
Levofloxacin	4	>16	≤0.06–>16	—	—	233	39.0	365	61.0	233	39.0	2	0.3	363	60.7
Mupirocin^a^	0.25	1	≤0.06–>256	543	90.8	—	—	55	9.2	—	—	—	—	—	—
Fusidic acid	0.12	2	≤0.03–>16	536	89.6	—	—	62	10.4	—	—	—	—	—	—
Erythromycin	>16	>16	≤0.06–>16	209	34.9	—	—	389	65.1	205	34.3	15	2.5	378	63.2
Clindamycin	0.12	>16	≤0.015–>16	474	79.3	—	—	124	20.7	476	79.6	5	0.8	117	19.6
Erythromycin/clidamycin	≤1/0.5	>8/1.5	≤1/0.5–>8/1.5	—	—	—	—	—	—	—	—	—	—	—	—
Retapamulin^a^	0.06	0.12	≤0.015–>2	590	98.7	—	—	8	1.3	—	—	—	—	—	—
All MSSA (*n* = 856)															
Penicillin	>0.5	>0.5	≤0.03–>0.5	215	25.1	—	—	641	74.9	215	25.1	—	—	641	74.9
Cefoxitin^a^	≤4	≤4	≤4	856	100	—	—	0	0	—	—	—	—	—	—
Ozenoxacin	0.002	0.004	≤0.001–>4	—	—	—	—	—	—	—	—	—	—	—	—
Vancomycin	1	1	≤0.25–>2	855	99.9	—	—	1	0.1	855	99.9	1	0.1	—	—
Ciprofloxacin	0.5	1	≤0.06–>16	—	—	774	90.4	82	9.6	774	90.4	25	2.9	57	6.7
Levofloxacin	0.25	0.5	≤0.06–>16	—	—	801	93.6	55	6.4	801	93.6	2	0.2	53	6.2
Mupirocin^a^	0.25	0.25	≤0.06–>256	833	97.3	—	—	23	2.7	—	—	—	—	—	—
Fusidic acid	0.12	0.25	≤0.03–>16	820	95.8	—	—	36	4.2	—	—	—	—	—	—
Erythromycin	0.25	>16	≤0.06–>16	573	66.9	—	—	283	33.1	570	66.6	19	2.2	267	31.2
Clindamycin	0.12	0.12	≤0.015–>16	823	96.1	—	—	33	3.9	825	96.4	0	0	31	3.6
Erythromycin/clidamycin	≤1/0.5	>8/1.5	≤1/0.5–>8/1.5	—	—	—	—	—	—	—	—	—	—	—	—
Retapamulin^a^	0.06	0.12	≤0.015–>2	852	99.5	—	—	4	0.5	—	—	—	—	—	—

Ciprofloxacin MIC values were interpreted as susceptible (*S* ≤ 0.001 mg/L; *I *= 0.001–1 mg/L) and resistant (*R* > 1 mg/L) according to the clinical breakpoints (EUCAST-2023).

No breakpoints are yet defined by the CLSI or EUCAST for ozenoxacin.

^a^MIC results were interpreted following the epidemiological cut-off (ECOFF) values (EUCAST-2023).

**Table 2.** Summary MIC data per year for ozenoxacin against *S. aureus* and *S. pyogenes* according to EUCAST-2023 guides and methicillin and ciprofloxacin susceptibility

	2020			2021			2022			Total (2020–2022)					
	Number	MIC_50_/MIC_90_	Percentage of inhibition^c^	Number	MIC_50_/MIC_90_	Percentage of inhibition^c^	Number	MIC_50_/MIC_90_	Percentage of inhibition^c^	Number	MIC_50_/MIC_90_	Percentage of inhibition^c^	MUP-R (%)	FUS-R (%)	RET-R (%)
All *S. aureus*	527	0.002/0.12	98.9	508	0.002/0.12	97.8	419	0.002/0.06	99.0	1454	0.002/0.12	98.6	5.4	6.7	0.8
All *S. aureus* CIP-R^a^	165	0.06/0.12	96.4	162	0.06/0.12	93.2	128	0.06/0.12	96.9	455	0.06/0.12	95.4	13	6.6	1.5
All *S. aureus* CIP-S^a^	362	0.002/0.002	100	346	0.002/0.004	100	291	0.002/0.002	100	999	0.002/0.004	100	1.9	6.8	0.5
All MRSA	212	0.06/0.12	97.6	217	0.06/0.12	94.9	169	0.06/0.12	98.8	598	0.06/0.12	97.0	9.2	10.4	1.3
All MRSA-CIP-R^a^	134	0.06/0.25	96.3	136	0.06/0.25	91.2	103	0.06/0.12	93.2	373	0.06/0.25	95.2	12.3	6.7	1.6
All MRSA-CIP-S^a^	78	0.002/0.002	100	81	0.002/0.004	100	66	0.002/0.002	100	225	0.002/0.004	100	4.0	16.4	0.9
All MSSA	315	0.002/0.004	99.7	291	0.002/0.004	99.7	250	0.002/0.004	99.2	856	0.002/0.004	99.6	2.7	4.2	0.5
All MSSA-CIP-R^a^	31	0.06/0.12	96.8	26	0.06/0.12	100	25	0.03/0.5	92.3	82	0.06/0.12	96.3	15.9	6.1	1.2
All MSSA-CIP-S^a^	284	0.002/0.002	100	265	0.002/0.004	100	225	0.002/0.002	100	774	0.002/0.004	100	1.3	4.0	0.4
All *S. pyogenes*	123	0.008/0.015	98.4	65	0.015/0.03	96.9	83	0.015/0.06	96.5	271	0.015/0.03	98.9	3.7	0	0.4
All *S. pyogenes* CIP-R^b^	3	−/−	—	3	−/−	—	17	0.06/0.12	88.2	23	0.06/0.06	91.3	4.3	0	0
All *S. pyogenes* CIP-S^b^	120	0.008/0.015	99.2	62	0.015/0.03	100	66	0.015/0.03	100	248	0.008/0.06	99.6	3.6	0	0.4

No breakpoints are yet defined by the CLSI or EUCAST for ozenoxacin.

MIC_50/90_, range and % of inhibition was only calculated in the subsets with a minimum of 10 isolates.

MIC values of comparators were interpreted as resistant as follows: mupirocin (*S. aureus*, MIC > 1 mg/L; *S. pyogenes*, MIC > 0.5 mg/L), fusidic acid (*S. aureus*, MIC > 1 mg/L; *S. pyogenes*, MIC > 16 mg/L) and retapamulin (*S. aureus*, MIC > 0.5 mg/L; *S. pyogenes*, MIC > 0.125 mg/L).

CIP-R, ciprofloxacin resistant; CIP-S, ciprofloxacin susceptible; MUP-R, mupirocin resistant; FUS-R, fusidic acid resistant; RET-R, retapamulin resistant

^a^For *S. aureus*, ciprofloxacin MIC values were interpreted as susceptible (*S *≤ 0.001 mg/L; *I* = 0.001–1 mg/L) and resistant (*R* > 1 mg/L) according to the clinical breakpoints (EUCAST-2023).

^b^For *S. pyogenes*, ciprofloxacin MIC values were interpreted as susceptible (*S* ≤ 1 mg/L) and resistant (*R* > 1 mg/L) according to the epidemiological cut-off (ECOFF) value (EUCAST-2023).

^c^Percentage of inhibition at MIC ≤ 0.5 mg/L in *S. aureus* and at MIC ≤0.06 mg/L in *S. pyogenes.*

Overall, ozenoxacin was the most potent agent. MIC_50_ value of ozenoxacin (MIC_50_ = 0.002 mg/L) was 5-fold serial dilution lower than that of retapamulin (MIC_50_ = 0.06 mg/L), 6-fold dilution greater than that of clindamycin and fusidic acid (MIC_50_ = 0.12 mg/L), 7-fold dilution greater than that of mupirocin, erythromycin and levofloxacin (MIC_50_ = 0.25 mg/L) and 8-fold dilution greater than that of ciprofloxacin (MIC_50_ = 0.5 mg/L). At the MIC_90_ level, ozenoxacin (MIC_90_ = 0.12 mg/L), was 1-fold dilution more active than fusidic acid (MIC_90_ = 0.25 mg/L), 2-fold dilution more active than mupirocin (MIC_90_ = 0.5 mg/L), 5-fold dilution more potent than clindamycin (MIC_90_ = 4 mg/L) and at least 7-fold dilution more active than erythromycin, ciprofloxacin or levofloxacin (MIC_90_ ≥ 16 mg/L for all) (Table 1).

The activity of ozenoxacin was higher against ciprofloxacin-susceptible *S. aureus* (MIC_50/90_ = 0.002/0.004 mg/L, range ≤0.001–0.5 mg/L) (100% MIC ≤0.5 mg/L) compared with that against ciprofloxacin-resistant isolates (MIC_50/90_ = 0.06/0.12 mg/L, range ≤0.001–>4 mg/L) (95.4% MIC ≤ 0.5 mg/L). Ozenoxacin was the most active compound against ciprofloxacin resistant *S. aureus* isolates (MIC_50_ = 0.06 mg/L; MIC_90_ = 0.12 mg/L) along with retapamulin (MIC_50_ = 0.06 mg/L, MIC_90_ = 0.12 mg/L). MIC_50_ value of ozenoxacin was 1-fold dilution greater than that of clindamycin and fusidic acid (MIC_50_ = 0.12 mg/L) and 2-fold dilution more potent than mupirocin (MIC_50_ = 0.25 mg/L). Comparative MIC_90_ data showed that the activity of ozenoxacin (MIC_90_ = 0.12 mg/L) was slightly higher than that of fusidic acid (MIC_90_ = 0.25 mg/L), but at least 7-fold dilution more active than mupirocin and clindamycin (MIC_90_ ≥ 16 mg/L). The remaining agents had higher MIC_50_ and MIC_90_ values than ozenoxacin (Table 1).

MRSA (*n* = 598) had slightly raised MIC compared to ozenoxacin (MIC_50/90_ = 0.06/0.12 mg/L, range ≤0.001–2 mg/L) than MSSA (*n* = 856) isolates (MIC_50/90_ = 0.002/0.004 mg/L, range ≤0.001–>4 mg/L). On the other hand, MRSA that was also ciprofloxacin resistant (*n* = 373) had an MIC_50/90_ of 0.06/0.25 mg/L (range 0.002–2 mg/L), slightly higher than that obtained in MSSA isolates that were ciprofloxacin resistant (*n* = 82) (MIC_50/90_ = 0.06/0.12 mg/L, range ≤0.001–>4 mg/L). Up to 99.6% of MSSA isolates showed an ozenoxacin MIC ≤ 0.5 mg/L, whereas 97.0% of MRSA were inhibited at a MIC ≤ 0.5 mg/L. Percentages of inhibition of ozenoxacin at a MIC ≤0.5 mg/L in ciprofloxacin-resistant MRSA and ciprofloxacin-resistant MSSA isolates were 95.2% and 96.3%, respectively (Table 2). Among the comparators, only retapamulin (MIC_50/90_ = 0.06/0.12 mg/L) and fusidic acid (MIC_50/90_ = 0.12/0.25 mg/L) showed comparable activity to that of ozenoxacin (MIC_50/90_ = 0.06/0.25 mg/L) in the subset of ciprofloxacin-resistant MRSA isolates. Resistance rates of mupirocin, fusidic acid and retapamulin in the subset of MRSA strains were 9.2%, 10.4% and 1.3%, respectively. The activity of the remaining agents is shown in Table S3.

### *Ozenoxacin* in vitro *activity in* S. pyogenes

**Figure 2.**
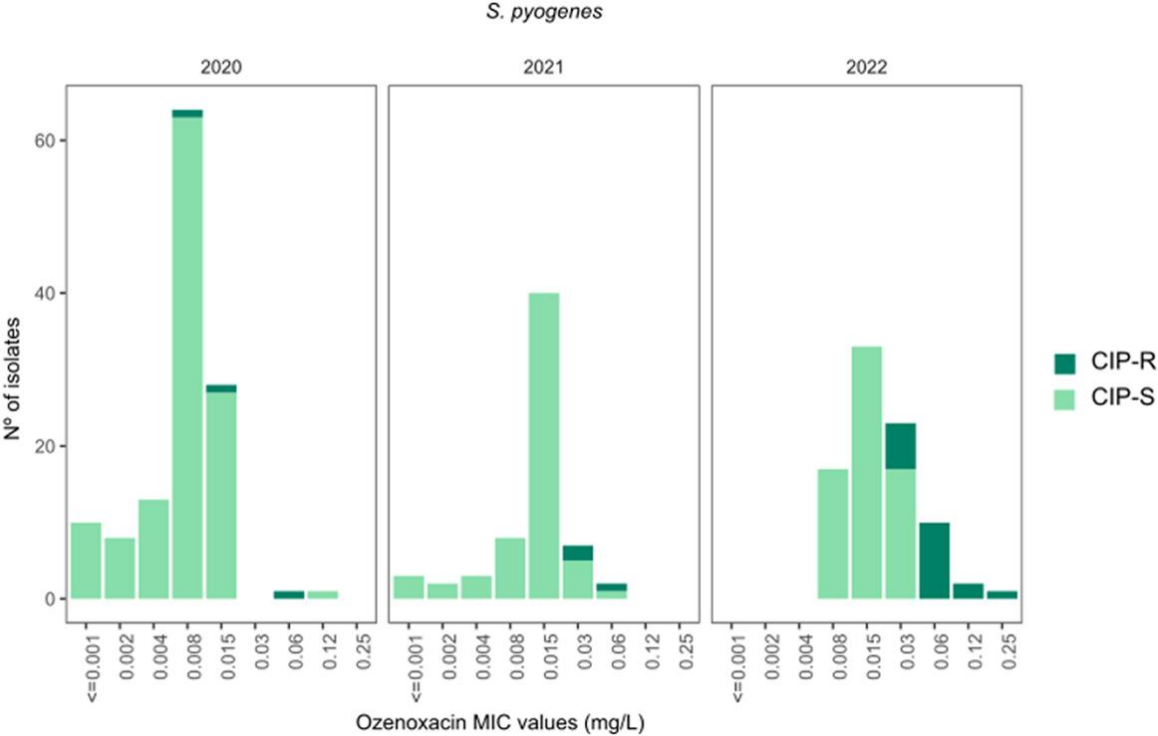
Distribution per year of the *S. pyogenes* isolates (2020, *n* = 123; 2021, *n* = 65; 2022, *n* = 85) by the ozenoxacin MIC value and the ciprofloxacin susceptibility. Ciprofloxacin MIC values were interpreted as susceptible (*S* ≤ 1 mg/L) and resistant (*R* > 1 mg/L) according to the epidemiological cut-off (ECOFF) value (EUCAST-2023).

According to both EUCAST and CLSI criteria, all *S. pyogenes* isolates (*n* = 271) were susceptible to penicillin, vancomycin and fusidic acid. Susceptibility rates of other antimicrobials according to EUCAST were as follows: retapamulin, 99.6%; levofloxacin, 98.5%; mupirocin, 96.3%; clindamycin, 95.9%; ciprofloxacin, 91.5% and erythromycin, 84.5%) (Table 3). A small increase in the ozenoxacin MIC_50/90_ values was observed over time: 2020, MIC_50/90_ = 0.008/0.015 mg/L, 99.2% MIC ≤ 0.06 mg/L; 2021, MIC_50/90_ = 0.015/0.03, 96.9% MIC ≤ 0.06 mg/L and 2022, MIC_50/90_ = 0.015/0.06 mg/L, 96.5% MIC ≤ 0.06 mg/L. Moreover, a higher number of isolates resistant to ciprofloxacin was also detected in 2022 (17/23) compared to the previous 2 years [2020 (3/23) and 2021 (3/23)] (Table 2, Figure 2).

**Table 3.** Antimicrobial activity of ozenoxacin and comparators tested against *S. pyogenes* by ciprofloxacin susceptibility

				EUCAST						CLSI					
	MIC (mg/mL)			S		I		R		S		I		R	
	MIC_50_	MIC_90_	Range	*n*	%	*n*	%	*n*	%	*n*	%	*n*	%	*n*	%
All *S. pyogenes* (*n* = 271)															
Penicillin	≤0.03	≤0.03	—	271	100	—	—	0	0	270	99.6	—	—	1	0.4
Cefoxitin	≤4	≤4	—	271	100	—	—	0	0	—	—	—	—	—	—
Ozenoxacin	0.015	0.03	≤0.001–0.12	—	—	—	—	—	—	—	—	—	—	—	—
Vancomycin	0.25	1	≤0.25–1	271	100	—	—	0	0	271	100	—	—	0	0
Ciprofloxacin^a^	0.5	1	≤0.06–8	—	—	248	91.5	23	8.5	—	—	—	—	—	—
Levofloxacin^a^	0.5	1	≤0.06–8	—	—	267	98.5	4	1.5	267	98.5	4	1.5	0	0
Mupirocin^a^	0.12	0.25	≤0.06–>256	261	96.3	—	—	10	3.7	—	—	—	—	—	—
Fusidic acid^a^	2	4	≤0.03–16	271	100	—	—	0	0	—	—	—	—	—	—
Erythromycin	≤0.06	4	≤0.06–>16	229	84.5	—	—	42	15.5	224	82.7	5	1.8	42	15.5
Clindamycin	≤0.03	0.12	≤0.015–>16	260	95.9	—	—	11	4.1	257	94.8	3	1.1	11	4.1
Erythromycin/clidamycin	≤1/0.5	>8/1.5	≤1/0.5–>8/1.5	—	—	—	—	—	—	—	—	—	—	—	—
Retapamulin^a^	≤0.015	0.03	≤0.015–>2	270	99.6	—	—	1	0.4	—	—	—	—	—	—
All *S. pyogenes* ciprofloxacin resistant (*n* = 23)															
Penicillin	≤0.03	≤0.03	—	23	100	—	—	0	0	23	100.0	—	—	0	0
Cefoxitin	≤4	≤4	—	23	100	—	—	0	0	—	—	—	—	—	—
Ozenoxacin	0.06	0.06	0.008–0.12	—	—	—	—	—	—	—	—	—	—	—	—
Vancomycin	0.5	1	≤0.25–1	23	100	—	—	0	0	23	100.0	—	—	0	0
Ciprofloxacin^a^	2	4	2–8	—	—	0	0	23	100	—	—	—	—	—	—
Levofloxacin^a^	2	4	1–8	—	—	19	82.6	4	17.4	19	82.6	4	17.4	0	0
Mupirocin^a^	0.25	0.5	≤0.06–>256	22	95.7	—	—	1	4.3	—	—	—	—	—	—
Fusidic acid^a^	4	8	1–8	23	100	—	—	0	0	—	—	—	—	—	—
Erythromycin	≤0.06	>16	≤0.06–>16	15	65.2	—	—	8	34.8	15	65.2	0	0	8	34.8
Clindamycin	≤0.03	0.12	≤0.015–>16	18	78.3	—	—	5	21.7	18	78.3	0	0	5	21.7
Erythromycin/clidamycin	≤1/0.5	>8/1.5	≤1/0.5–>8/1.5	—	—	—	—	—	—	—	—	—	—	—	—
Retapamulin^a^	0.06	0.06	≤0.015–0.12	23	100	—	—	0	0	—	—	—	—	—	—
All *S. pyogenes* ciprofloxacin susceptible (*n* = 248)															
Penicillin	≤0.03	≤0.03	—	248	100	—	—	0	0	247	99.6	—	—	1	0.4
Cefoxitin	≤4	≤4	—	248	100	—	—	0	0	—	—	—	—	—	—
Ozenoxacin	0.008	0.06	≤0.001–0.12	—	—	—	—	—	—	—	—	—	—	—	—
Vancomycin	0.5	1	≤0.25–1	248	100	—	—	0	0	248	100	—	—	0	0
Ciprofloxacin^a^	2	4	2–8	—	—	248	100	0	0	—	—	—	—	—	—
Levofloxacin^a^	2	4	1–8	—	—	248	100	0	0	248	100	0	0	0	0
Mupirocin^a^	0.25	0.5	≤0.06–>256	239	96.4	—	—	9	3.6	—	—	—	—	—	—
Fusidic acid^a^	4	8	1–8	248	100	—	—	0	0	—	—	—	—	—	—
Erythromycin	≤0.06	>16	≤0.06–>16	214	86.3	—	—	34	13.7	209	84.3	5	2.0	34	13.7
Clindamycin	≤0.03	0.12	≤0.015–>16	242	97.6	—	—	6	2.4	239	96.4	3	1.2	6	2.4
Erythromycin/clidamycin	≤1/0.5	>8/1.5	≤1/0.5–>8/1.5	—	—	—	—	—	—	—	—	—	—	—	—
Retapamulin^a^	≤0.015	0.03	≤0.015–>2	247	99.6	—	—	1	0.4	—	—	—	—	—	—

Ciprofloxacin MIC values were interpreted as susceptible (*S* ≤ 1 mg/L) and resistant (*R* > 1 mg/L) according to the epidemiological cut-off (ECOFF) value (EUCAST-2023).

No breakpoints are yet defined by the CLSI or EUCAST for ozenoxacin.

^a^MIC results were interpreted following the ECOFF values of EUCAST.

At the MIC_50_ level, ozenoxacin demonstrated potent activity against all *S. pyogenes* (MIC_50_ = 0.015 mg/L). MIC_50_ values for the other comparators tested were: retapamulin, MIC_50_ ≤ 0.015 mg/L; clindamycin, MIC_50_ = 0.03 mg/L; erythromycin, MIC_50_ ≤ 0.06 mg/L; mupirocin, MIC_50_ = 0.12 mg/L; vancomycin, MIC_50_ ≤ 0.25 mg/L; ciprofloxacin, MIC_50_ = 0.5 mg/L; levofloxacin, MIC_50_ = 0.5 mg/L and fusidic acid, MIC_50_ = 2 mg/L. Comparative MIC data showed that ozenoxacin activity (MIC_90_ = 0.03 mg/L) was equal to that of retapamulin (MIC_90_ = 0.03 mg/L). Nevertheless, ozenoxacin was 2-fold dilution more active than clindamycin (MIC_90_ = 0.12 mg/L); 3-fold dilution more active than mupirocin (MIC_90_ = 0.25 mg/L); 5-fold dilution more active than vancomycin (MIC_90_ = 1 mg/L), ciprofloxacin (MIC_90_ = 1 mg/L) and levofloxacin (MIC_90_ = 1 mg/L), and 7-fold dilution more active than erythromycin (MIC_90_ = 4 mg/L) and fusidic acid (MIC_90_ = 4 mg/L) (Table 3).

In the subset of ciprofloxacin resistant *S. pyogenes* isolates (*n* = 23), susceptibility rates by EUCAST were: penicillin, 100%; vancomycin, 100%; fusidic acid, 100%; retapamulin, 100%; clindamycin, 78.3%; mupirocin, 95.7% and erythromycin, 65.2%. Ozenoxacin also showed the highest activity (MIC_50/90_ = 0.06/0.06 mg/L, range 0.008–0.12 mg/L) along with retapamulin (MIC_50/90_ = 0.06/0.06 mg/L, range 0.008–0.12 mg/L). At MIC_50_ level, ozenoxacin (MIC_50_ = 0.06 mg/L) was comparable to erythromycin (MIC_50_ ≤ 0.06 mg/L) and clindamycin (MIC_50_ ≤ 0.03 mg/L), but was 2-fold dilutions more potent than mupirocin (MIC_50_ = 0.25 mg/L), 3-fold dilutions more active than vancomycin (MIC_50_ = 0.5 mg/L) and 6-fold dilutions more active than fusidic acid (MIC_50_ = 4 mg/L). At MIC_90_ level, ozenoxacin (MIC_90_ = 0.06 mg/L) was 1-fold dilution more active than clindamycin (MIC_90_ = 0.12 mg/L), 3-fold dilution more active than mupirocin (MIC_90_ = 0.5 mg/L), 4-fold dilution more active than vancomycin (MIC_90_ = 1 mg/L), 7-fold dilution more active than fusidic acid (MIC_90_ = 8 mg/L) and at least 9-fold dilution more potent than erythrodmycin (MIC_90_ > 16 mg/L) (Table 3).

## Discussion

In the present study, we compared the *in vitro* activity of ozenoxacin against recent clinical isolates (2020–2022) of MSSA, MRSA and *S. pyogenes*, including both quinolone-susceptible and quinolone-resistant isolates, with that of topical agents most frequently used for the treatment of superficial skin infections, including impetigo. *S. aureus* was the most frequent microorganism in our collection (85%), most of them from infection samples of community origin. Up to 41% of the *S. aureus* were resistant to methicillin and more than a half of them were also resistant to ciprofloxacin (62% of MRSA).

Confirming the results of previously published comparative studies, ozenoxacin exhibited potent *in vitro* activity against both *S. aureus* (MIC_50/90_ = 0.002/0.12 mg/L) and *S. pyogenes* (MIC_50/90_ = 0.015/0.03 mg/L), inhibiting 99% of the isolates at MIC ≤ 0.5 mg/L and at MIC ≤ 0.06, respectively.^12,16,17^ A previous multicentre study comparing two collections of Gram-positive clinical samples from 2009–2010 and 2014 showed similar ozenoxacin MIC values in both *S. aureus* (MIC_50/90_ = 0.004/0.25 mg/L in 2009–2010; MIC_50/90_ = 0.002/0.06 mg/L in 2014) and *S. pyogenes* (MIC_50/90_ = 0.03/0.06 mg/L in 2009–2010; MIC_50/90_ = 0.008/0.015 mg/L in 2014).^16^ Also in agreement with these studies, the more active comparator agent against *S. aureus* isolates was retapamulin (MIC_90_ = 0.12 mg/L), followed by fusidic acid (MIC_90_ = 0.25 mg/L) and mupirocin (MIC_90_ = 0.5 mg/L). Other agents with activity against *S. pyogenes* were retapamulin (MIC_90_ =0.03 mg/L), clindamycin (MIC_90_ = 0.12 mg/L) and mupirocin (MIC_90_ = 0.25 mg/L). These isolates also showed susceptible but with increased MIC_90_ values (MIC_90_ = 4 mg/L) of fusidic acid. Note that we observed a small increase over time in the ozenoxacin MIC_50/90_ values of *S. pyogenes* (from 0.008/0.015 mg/L in 2020 to 0.015/0.06 mg/L in 2022), possibly due to the increase of ciprofloxacin-resistant isolates in the last year.

As in previous studies, ozenoxacin maintained robust activity against all isolates, regardless of ciprofloxacin susceptibility.^12,16–18^ However, coinciding with these studies, compared with that observed in *S. aureus* isolates susceptible to ciprofloxacin (MIC_50/90_ = 0.002/0.004 mg/L), ozenoxacin had lower activity when tested against *S. aureus* resistant to ciprofloxacin (MIC_50/90_ = 0.06/0.12 mg/L). In *S. pyogenes*, ozenoxacin also showed higher activity against ciprofloxacin susceptible (MIC_50/90_ = 0.008/0.06 mg/L) than against ciprofloxacin resistant (MIC_50/90_ = 0.06/0.06 mg/L) isolates. Nevertheless, in both species the activity of ozenoxacin remained superior to that of the comparator agents against ciprofloxacin-resistant isolates and only retapamulin showed MIC_90_ values equal to those of ozenoxacin. Several studies have demonstrated excellent antibacterial activity of ozenoxacin against Gram-positive bacteria resistant to other quinolones, including strains carrying mutations in *gyrA* and/or *parC* genes, and low capacity to select resistant mutant strains.^18,21^ This could be explained by the different mechanisms of action of these agents. Ciprofloxacin acts preferentially against topoisomerase IV, whereas ozenoxacin has been shown to simultaneously inhibit DNA gyrase and topoisomerase IV at lower concentrations compared to other quinolones.^12^ Moreover, the efficacy of ozenoxacin is not affected in strains with active efflux systems, providing an advantage over other related agents.^12,13^

On the other hand, ozenoxacin was the most potent antimicrobial agent tested overall against MSSA isolates (MIC_50/90_ = 0.002/0.004 mg/L). As in previous studies, MRSA isolates had slightly increased MICs to ozenoxacin (MIC_50/90_ = 0.06/0.12 mg/L).^12,16,17^ This is probably due to a slight decrease in interaction with topoisomerases (cross-resistance) rather than methicillin-susceptibility; MRSA with susceptible ciprofloxacin MIC values displayed an ozenoxacin MIC_90_ of 0.004 mg/L. In fact, ozenoxacin exhibited higher activity than all comparator agents against MSSA isolates, while in the subsets of MRSA and ciprofloxacin resistance, activity of ozenoxacin was comparable to that of retapamulin and fusidic acid, and higher than that of the other compounds.

According to previous data, resistance to both mupirocin and fusidic acid has been documented in Europe and is increasing, particularly in community-associated MRSA infections.^8,10,11^ In addition, a low-level of resistance to retapamulin has also been described.^7,9^ In our MRSA collection, we found higher resistance rates to mupirocin (9%), fusidic acid (10%) and retapamulin (1.3%) than a previous study performed in China in 2020 (5%, 1% and 0.3%, respectively). These data underscore the need for alternative solutions and raise ozenoxacin as a valuable and safe alternative for the treatment of SSTIs, including impetigo. In this sense, a recent study in Canada showed an excellent activity of ozenoxacin against MSSA and MRSA isolates with high-level of resistance to mupirocin and fusidic acid.^17^ Note that both mupirocin and fusidic acid are bacteriostatic antibiotics while ozenoxacin shows a bactericidal activity.

The main limitation of our study is the absence of established breakpoints for ozenoxacin by both CLSI and EUCAST. Clinical breakpoints are not determined for topical antibiotics as PK/PD targets are not also defined. This underscores the need for continued surveillance and analysis of MIC distributions.

In conclusion, our study provides a temporal perspective of ozenoxacin susceptibility, revealing stability in MIC values over the 3 years of study. Our results highlight the consistent and potent activity of ozenoxacin against *S. aureus*, irrespective of their methicillin and ciprofloxacin resistance status. This potent activity was also observed in *S. pyogenes*, even when exhibiting ciprofloxacin resistance. Notably, ozenoxacin demonstrated superiority over comparator agents, including in the subset of ciprofloxacin-resistant MRSA isolates. Ongoing monitoring of antimicrobial resistance is crucial for informed decision making in empirical therapy, especially in the ever-evolving landscape of bacterial resistance patterns.

## Supplementary Material

dlae088_Supplementary_Data

## Supplementary data

Tables S1 to S3 are available as Supplementary data at *JAC-AMR* Online.
