# Supplementary material for: In vitro activity of ozenoxacin against Staphylococcus aureus and Streptococcus pyogenes clinical isolates recovered in a worldwide multicentre study (2020–2022)
Source: JAC Antimicrob Resist. 2024 Jun 13;6(3):dlae088. doi: 10.1093/jacamr/dlae088 (PMC11170484; doi:10.1093/jacamr/dlae088)
Supplement: dlae088_Supplementary_Data [file dlae088_supplementary_data.docx]

| **Table S1**. Distribution of isolates by microorganism and year of collection. | | | | | | | | |
| --- | --- | --- | --- | --- | --- | --- | --- | --- |
| **Microorganism / Year** | **2020** | | **2021** | | **2022** | | **TOTAL** | |
|  | **N** | **%** | **N** | **%** | **N** | **%** | **N** | **%** |
| **All *S. aureus*** | 527 | 30.6 | 508 | 29.4 | 419 | 24.3 | 1454 | 84.3 |
| Ciprofloxacin resistant | 165 | 9.6 | 162 | 9.4 | 128 | 7.4 | 455 | 26.4 |
| Ciprofloxacin susceptible | 362 | 21.0 | 346 | 20.1 | 291 | 16.9 | 999 | 57.9 |
| **MRSA** | 212 | 12.3 | 217 | 12.6 | 169 | 9.8 | 598 | 34.7 |
| Ciprofloxacin resistant | 78 | 4.5 | 81 | 4.7 | 66 | 3.8 | 225 | 13.0 |
| Ciprofloxacin susceptible | 134 | 7.8 | 136 | 7.9 | 103 | 6.0 | 373 | 21.6 |
| **MSSA** | 315 | 18.3 | 291 | 16.9 | 250 | 14.5 | 856 | 49.6 |
| Ciprofloxacin resistant | 284 | 16.5 | 265 | 15.4 | 225 | 13.0 | 774 | 44.9 |
| Ciprofloxacin susceptible | 31 | 1.8 | 26 | 1.5 | 25 | 1.4 | 82 | 4.8 |
| **All *S. pyogenes*** | 123 | 7.1 | 65 | 3.8 | 83 | 4.8 | 271 | 15.7 |
| Ciprofloxacin resistant | 3 | 0.2 | 3 | 0.2 | 17 | 1.0 | 23 | 1.3 |
| Ciprofloxacin susceptible | 120 | 7.0 | 62 | 3.6 | 66 | 3.8 | 248 | 14.4 |

| **Table S2.** Distribution of isolates by microorganism, type of skin infection and source of sample. | | | | | | | | | | | | | |
| --- | --- | --- | --- | --- | --- | --- | --- | --- | --- | --- | --- | --- | --- |
|  | ***S. aureus*** | | | | | ***S. pyogenes*** | | | | | | **TOTAL** | |
| **Source of sample/ type of infection** | **Community acquired** |  | **Other** | **Unknown** | **TOTAL** | | **Community acquired** |  | **Other** | **Unknown** | **TOTAL** | |  |
| **Abcess** | 235 |  | 19 | 11 | 265 | | 54 |  | 0 | 5 | 59 | | **324** |
| **Wound** | 505 |  | 43 | 49 | 597 | | 92 |  | 4 | 15 | 111 | | **708** |
| **Soft tissue** | 124 |  | 6 | 53 | 183 | | 15 |  | 0 | 7 | 22 | | **205** |
| **Skin** | 162 |  | 41 | 64 | 277 | | 50 |  | 1 | 25 | 76 | | **353** |
| **Other** | 16 |  | 0 | 2 | 18 | | 2 |  | 0 | 1 | 3 | | **21** |
| **Unknown** | 39 |  | 0 | 75 | 114 | | 0 |  | 0 | 0 | 0 | | **114** |
| **Total** | 1091 |  | 109 | 254 | **1454** | | 213 |  | 5 | 53 | **271** | | **1725** |

*According to the EMA, skin and soft tissue infections (SSTI) include cellulitis, erysipelas, wound infections (traumatic or post-surgical) and major abscesses.

| **Table S3.** Antimicrobial activity of ozenoxacin and comparators tested against MRSA and MSSA isolates by ciprofloxacin susceptibility. | | | | | | | | | | | | | | | |
| --- | --- | --- | --- | --- | --- | --- | --- | --- | --- | --- | --- | --- | --- | --- | --- |
|  | **^a^MIC (mg/mL)** | | | **EUCAST** | | | | | | **CLSI** | | | | | |
|  |  |  |  | **S** | | **I** | | **R** | | **S** | | **I** | | **R** | |
|  | **MIC50** | **MIC90** | **Range** | **N** | **%** | **N** | **%** | **N** | **%** | **N** | **%** | **N** | **%** | **N** | **%** |
| **All MRSA-CIP-S (n=225)** |  |  |  |  |  |  |  |  |  |  |  |  |  |  |  |
| Penicillin | >0.5 | >0.5 | ≤0.03->0.5 | 2 | 0.9 | - | - | 223 | 99.1 | 2 | 0.9 | - | - | 223 | 99.1 |
| Cefoxitin* | >4 | >4 | >4 | 0 | 0 | - | - | 225 | 100 | - | - | - | - | - | - |
| Ozenoxacin | 0.002 | 0.004 | ≤0.001-0.03 | - | - | - | - | - | - | - | - | - | - | - | - |
| Vancomycin | 1 | 1 | ≤0.25->2 | 224 | 99.6 | - | - | 1 | 0.4 | 224 | 99.6 | 1 | 0.4 | - | - |
| Ciprofloxacin | 0.25 | 0.5 | 0.12-1 | - | - | 225 | 100 | 0 | 0 | 225 | 100 | 0 | 0 | 0 | 0 |
| Levofloxacin | 0.25 | 0.25 | ≤0.06-8 | - | - | 224 | 99.6 | 1 | 0.4 | 224 | 99.6 | 0 | 0 | 1 | 0.4 |
| Mupirocin* | 0.25 | 0.25 | 0.12->256 | 216 | 96.0 | - | - | 9 | 4.0 | - | - | - | - | - | - |
| Fusidic acid | 0.12 | 8 | ≤0.03->16 | 188 | 83.6 | - | - | 37 | 16.4 | - | - | - | - | - | - |
| Erythromycin | 0.25 | >16 | ≤0.06->16 | 123 | 54.7 | - | - | 102 | 45.3 | 122 | 54.2 | 6 | 2.7 | 97 | 43.1 |
| Clindamycin | 0.12 | 8 | 0.03->16 | 197 | 87.6 | - | - | 28 | 12.4 | 198 | 88.0 | 3 | 1.3 | 24 | 10.7 |
| Erythromycin/clidamycin | ≤1/0.5 | >8/1.5 | ≤1/0.5->8/1.5 | - | - | - | - | - | - | - | - | - | - | - | - |
| Retapamulin* | 0.06 | 0.12 | 0.03->2 | 223 | 99.1 | - | - | 2 | 0.9 | - | - | - | - | - | - |
|  |  |  |  |  |  |  |  |  |  |  |  |  |  |  |  |
| **All MRSA-CIP-R (n=373)** |  |  |  |  |  |  |  |  |  |  |  |  |  |  |  |
| Penicillin | >0.5 | >0.5 | ≤0.03->0.5 | 2 | 0.5 | - | - | 371 | 99.5 | 2 | 0.5 | - | - | 371 | 99.5 |
| Cefoxitin* | >4 | >4 | >4 | 0 | 0 | - | - | 373 | 100 | - | - | - | - | - | - |
| Ozenoxacin | 0.06 | 0.25 | 0.002-2 | - | - | - | - | - | - | - | - | - | - | - | - |
| Vancomycin | 1 | 1 | ≤0.25->2 | 371 | 99.5 | - | - | 2 | 0.5 | 371 | 99.5 | 2 | 0.5 | - | - |
| Ciprofloxacin | >16 | >16 | 2->16 | - | - | 0 | 0 | 373 | 100 | 0 | 0 | 8 | 2.1 | 365 | 97.9 |
| Levofloxacin | 8 | 16 | 0.5->16 | - | - | 9 | 2.4 | 364 | 97.6 | 9 | 2.4 | 2 | 0.5 | 362 | 97.1 |
| Mupirocin* | 0.25 | 8 | ≤0.06->256 | 327 | 87.7 | - | - | 46 | 12.3 | - | - | - | - | - | - |
| Fusidic acid | 0.12 | 0.25 | ≤0.03->16 | 348 | 93.3 | - | - | 25 | 6.7 | - | - | - | - | - | - |
| Erythromycin | >16 | >16 | ≤0.06->16 | 86 | 23.1 | - | - | 287 | 76.9 | 83 | 22.3 | 9 | 2.4 | 281 | 75.3 |
| Clindamycin | 0.12 | >16 | ≤0.015->16 | 277 | 74.3 | - | - | 96 | 25.7 | 278 | 74.5 | 2 | 0.5 | 93 | 24.9 |
| Erythromycin/clidamycin | ≤1/0.5 | >8/1.5 | ≤1/0.5->8/1.5 | - | - | - | - | - | - | - | - | - | - | - | - |
| Retapamulin* | 0.06 | 0.12 | ≤0.015->2 | 367 | 98.4 | - | - | 6 | 1.6 | - | - | - | - | - | - |
|  |  |  |  |  |  |  |  |  |  |  |  |  |  |  |  |
| **All MSSA-CIP-S (n=774)** |  |  |  |  |  |  |  |  |  |  |  |  |  |  |  |
| Penicillin | >0.5 | >0.5 | ≤0.03->0.5 | 198 | 25.6 | - | - | 576 | 74.4 | 198 | 25.6 | - | - | 576 | 74.4 |
| Cefoxitin* | ≤4 | ≤4 | ≤4 | 774 | 100 | - | - | 0 | 0 | - | - | - | - | - | - |
| Ozenoxacin | 0.002 | 0.004 | ≤0.001-0.5 | - | - | - | - | - | - | - | - | - | - | - | - |
| Vancomycin | 1 | 1 | ≤0.25-2 | 774 | 100 | - | - | 0 | 0 | 774 | 100 | 0 | 0 | - | - |
| Ciprofloxacin | 0.25 | 0.5 | ≤0.06-1 | - | - | 774 | 100 | 0 | 0 | 774 | 100 | 0 | 0 | 0 | 0 |
| Levofloxacin | 0.25 | 0.25 | ≤0.06-1 | - | - | 774 | 100 | 0 | 0 | 774 | 100 | 0 | 0 | 0 | 0 |
| Mupirocin* | 0.25 | 0.25 | ≤0.06->256 | 764 | 98.7 | - | - | 10 | 1.3 | - | - | - | - | - | - |
| Fusidic acid | 0.12 | 0.25 | ≤0.03->16 | 743 | 96.0 | - | - | 31 | 4.0 | - | - | - | - | - | - |
| Erythromycin | 0.25 | >16 | ≤0.06->16 | 529 | 68.3 | - | - | 245 | 31.7 | 526 | 68.0 | 17 | 2.2 | 231 | 29.8 |
| Clindamycin | 0.12 | 0.12 | ≤0.015->16 | 757 | 97.8 | - | - | 17 | 2.2 | 759 | 98.1 | 0 | 0 | 15 | 1.9 |
| Erythromycin/clidamycin | ≤1/0.5 | >8/1.5 | ≤1/0.5->8/1.5 | - | - | - | - | - | - | - | - | - | - | - | - |
| Retapamulin* | 0.06 | 0.12 | ≤0.015->2 | 771 | 99.6 | - | - | 3 | 0.4 | - | - | - | - | - | - |
|  |  |  |  |  |  |  |  |  |  |  |  |  |  |  |  |
| **All MSSA-CIP-R (n=82)** |  |  |  |  |  |  |  |  |  |  |  |  |  |  |  |
| Penicillin | >0.5 | >0.5 | ≤0.03->0.5 | 17 | 20.7 | - | - | 65 | 79.3 | 17 | 20.7 | - | - | 65 | 79.3 |
| Cefoxitin* | ≤4 | ≤4 | ≤4 | 82 | 100 | - | - | 0 | 0 | - | - | - | - | - | - |
| Ozenoxacin | 0.06 | 0.12 | ≤0.001->4 | - | - | - | - | - | - | - | - | - | - | - | - |
| Vancomycin | 1 | 1 | ≤0.25->2 | 81 | 98.8 | - | - | 1 | 1.2 | 81 | 98.8 | 1 | 1.2 | - | - |
| Ciprofloxacin | 8 | >16 | 2->16 | - | - | 0 | 0 | 82 | 100 | 0 | 0 | 25 | 30.5 | 57 | 69.5 |
| Levofloxacin | 4 | >16 | 0.5->16 | - | - | 27 | 32.9 | 55 | 67.1 | 27 | 32.9 | 2 | 2.4 | 53 | 64.6 |
| Mupirocin* | 0.25 | >256 | ≤0.06->256 | 69 | 84.1 | - | - | 13 | 15.9 | - | - | - | - | - | - |
| Fusidic acid | 0.12 | 0.25 | ≤0.03->16 | 77 | 93.9 | - | - | 5 | 6.1 | - | - | - | - | - | - |
| Erythromycin | 0.5 | >16 | ≤0.06->16 | 44 | 53.7 | - | - | 38 | 46.3 | 44 | 53.7 | 2 | 2.4 | 36 | 43.9 |
| Clindamycin | 0.12 | >16 | 0.06->16 | 66 | 80.5 | - | - | 16 | 19.5 | 66 | 80.5 | 0 | 0 | 16 | 19.5 |
| Erythromycin/clidamycin | ≤1/0.5 | >8/1.5 | ≤1/0.5->8/1.5 | - | - | - | - | - | - | - | - | - | - | - | - |
| Retapamulin* | 0.06 | 0.12 | ≤0.015->2 | 81 | 98.8 | - | - | 1 | 1.2 | - | - | - | - | - | - |

*MIC results were interpreted following the ECOFF values of EUCAST.

No breakpoints are yet defined by the CLSI or EUCAST for ozenoxacin.
